# Treatment of membranous nephropathy with crescent nephritis by rituximab: A case report

**DOI:** 10.1097/MD.0000000000030663

**Published:** 2022-09-16

**Authors:** Fan Zhang, Yiya Yang, Yinyin Chen, Ying Chen, Wei Yin, Yumei Liang, Xun Luo

**Affiliations:** a Department of Nephrology and Laboratory of Kidney Disease, Hunan Provincial People’s Hospital, Hunan Normal University, Changsha, China.

**Keywords:** antiphospholipaseA2 receptor antibodies, crescentic glomerulonephritis, membranous nephropathy

## Abstract

Crescent formation is rare in primary membranous nephropathy (MN). The mechanism of crescent formation is unknown and the treatments are tentative.

**Patient concerns:**

A 71-year-old woman presented with nephrotic syndrome, hematuria, and rapidly progressive kidney dysfunction.

**Diagnosis:**

Kidney biopsy was performed, and the diagnosis was MN in combination with crescentic glomerulonephritis. Circulating anti-PLA2R was detected of a high level.

**Interventions:**

The patient received rituximab besides corticosteroids.

**Outcomes:**

The patient achieved complete remission of proteinuria and recovery of kidney function.

**Conclusion:**

Our case suggests that there is a pathologic feature of MN and crescents in the absence of known immunologic factors as well as rituximab could serve as an effective cure and could be considered in serious MN conditions.

## Introduction

Membranous nephropathy (MN) is a major cause of nephrotic syndrome(NS) in adults.^[1,2]^ It may be primary(idiopathic) or secondary to systemic autoimmune or other diseases, such as neoplasms, chronic infections, or drugs.^[1,3]^ Kidney histomorphology shows a thickened glomerular basement membrane (GBM), granular staining of IgG and complement along the periphery of glomerular capillary loops, and electron-dense subepithelial deposits.^[4]^ Phospholipase A2 receptor (PLA2R) on podocytes is the major autoantigen.^[5,6]^ In most cases of primary MN, antibodies directed against PLA2R are detected in the plasma or kidney tissue, and studies have shown that the titer of anti-PLA2R antibodies is correlated with urinary protein excretion and disease activity. Antibodies may disappear during spontaneous or treatment-induced remission and may reoccur at relapse. A high antibody level is associated with a lower chance of remission and a higher risk of renal function deterioration.^[7–9]^

The crescent formation is rare in primary membranous nephropathy (MN). Crescentic glomerulonephritis usually occurs in the presence of anti-GBM antibodies, antineutrophil cytoplasmic antibodies (ANCA), lupus nephritis, or IgA nephropathy.^[10]^ The combination of MN and crescentic glomerulonephritis is rare. Previously reported cases of MN and crescent formation without any signs of vasculitis, lupus, or anti-GBM disease showed unfavorable therapeutic responses and tended to have worse renal outcomes. The mechanism of crescent formation is unknown, and treatments are tentative.

Here, we present a rare case of NS and kidney biopsy-proven MN. High levels of anti-PLA2R were detected in the circulation. Corticosteroids and rituximab treatments led to complete remission of both proteinuria and kidney dysfunction, which implies that rituximab could serve as an effective cure and could be considered in serious conditions of MN.

## Case description

A 71-year-old woman was admitted to our hospital with nausea, vomiting, and hypourocrinia for 20 days. Twenty days before admission, she presented with nausea, vomiting, and urine volume decreased to 200 mL/d. At initiation, serum creatinine and electrolyte levels were within the normal range, and the serum creatinine increased to 224 µmol/L then 539 µmol/L before admission to our hospital. Urinary sediment showed 120,000 red blood cells per mL, 4+ protein, and serum albumin was 24.99 g/L. His serum creatinine was 547 µmol/L. She had a history of hypertension and type 2 diabetes. Her serum creatinine was 86 mmol/L 4 months ago. On admission, her temperature was 36.0°C, her blood pressure was 153/77 mmHg, and her heart rate was 71 beats per minute. Physical examination was unremarkable. CT showed pulmonary interstitial inflammation and, bilateral pleural effusion, and ultrasound showed kidney size enlarged. Anti-PLA2R antibodies were positive at 415.29(<14) RU/mL. ANCA, anti-GBM antibody, anti-PR3 antibody, and ANA antibody were all negative. Complement C3 and C4 were normal. Immunofixation electrophoresis of blood was negative. Hepatitis B, hepatitis C, syphilis, and HIV screening results were negative.

**Figure 1.**
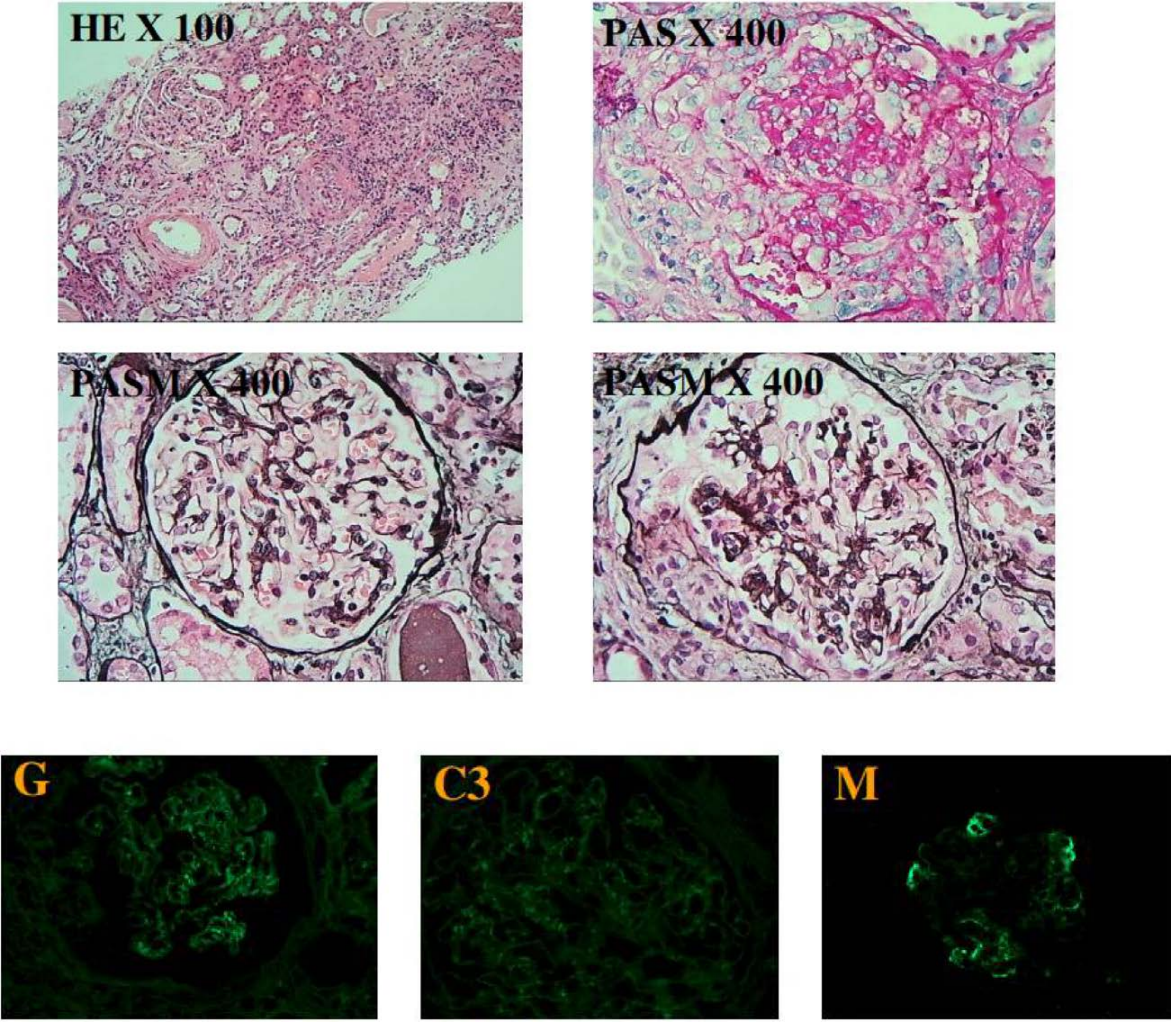
Kidney biopsy examinations. By light microscopy, focal glomerulus shows cellular crescents and thickened glomerular basement membranes (GBMs). Immunofluorescence study showed granular deposits of IgG++(E), C3+(F), and IgM+(G) along capillary loop.

**Figure 2.**
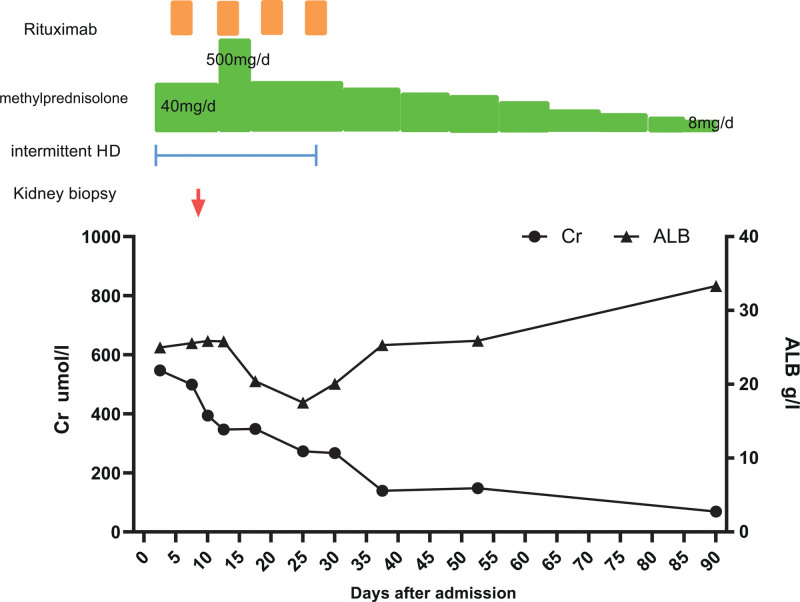
Clinical course of the patient. ALB = albumin, Cr = creatinine, HD = hemodialysis.

Kidney biopsy (Fig. 1) contained 11 glomeruli,1 of them were global sclerosis and 6 of them had crescent formation, including 2 fibrocellularcrescents,1 small cellularcrescents, and 1 small fibrocellularcrescents.GBM thickening and “spike and dome” appearance were observed. Renal tubules presented with epithelial cells granular degeneration with few protein casts. The interstitium was infiltered by inflammatory cells and mild fibrosis. Immunofluorescence revealed granular deposits of IgG++, C3+, and IgM+ along the capillary loop, and the diagnosis was stage II MN combined with crescentic glomerulonephritis. She was treated with methylprednisolone 500 mg per day for 3 days and then reduced to 40 mg per day, in combination with rituximab 600 mg per week for 4 weeks (total dose 2.4 mg), combined with intermittent hemodialysis. One month later, the patient achieved partial remission of proteinuria and recovery of renal function (Fig. 2). Her serum creatinine level was 139 µmol/L (range, 40–100 µmol/L). Urinary sediment showed no red blood cells and 2+ protein. Glucocorticoids were gradually reduced, and the patient achieved complete remission 3 months later.Anti-PLA2R antibody testing became negative.

## Discussion

This case report describes a crescentic MN presenting with NS and a rapid decline in Glomerular filtration rate (GFR), which showed a good response to new immunosuppressive treatment with corticosteroids and the anti-CD20 monoclonal antibody rituximab. Crescent formation is a rare complication of MN in the absence of systemic autoimmune disease or chronic infection and is usually associated with ANCA-positive vasculitis, anti-GBM nephritis, lupus, or IgA nephropathy. Investigators have proposed that the pathogenicity of anti-PLA2R antibodies may play a crucial role in the crescent formation of MN lacking anti-GBM, ANCA, or lupus.^[11]^ These cases are limited to very few reports in the literature.^[12]^

According to previously reported cases,^[12]^ our case presented with similar clinical features, kidney biopsy findings, and laboratory results, with glomeruli showing, on average, 25% (range, 2%–73%) involvement by crescents. Previously reported patients who received immunosuppressive therapy or angiotensin-converting enzyme inhibitors(ACEI)/angiotensin receptor blockers(ARBs) showed poor response to therapy and progressed to end-stage renal disease or deteriorated renal function.^[12,13]^ Currently no recommendations or guidelines are available for the treatment of MN with crescents. This patient achieved complete remission of proteinuria and recovery of kidney function with the combined use of corticosteroids and rituximab. Rituximab is a murine/human chimeric anti-CD20 monoclonal antibody, that has been applied in the treatment of membranous nephropathy and has achieved good results. For refractory MN cases, rituximab has been documented as a successful treatment option.

## Conclusion

In summary, our case suggests a beneficial effect of rituximab accompanied by corticosteroids in patients with MN and crescentic glomerulonephritis. It also suggests that there is a pathologic feature of MN and crescents in the absence of known immunologic factors, and rituximab could serve as an effective cure and could be considered in serious MN conditions.

## Author contributions

Conceptualization: Fan Zhang,Yumei Liang,Xun Luo.

Data curation: Fan Zhang,Yiya Yang.

Investigation:Ying Chen,Wei Yin.

Supervision: Yumei Liang,Xun Luo.

Writing – original draft: Fan Zhang.

Writing – review & editing:Yinyin Chen.
